# Pleural malakoplakia caused by Rhodoccocus equi infection in a patient after stem cell transplantation

**DOI:** 10.1186/1746-1596-7-20

**Published:** 2012-02-23

**Authors:** Carl Ludwig Behnes, Silke Neumann, Stefan Schweyer, Heinz-Joachim Radzun

**Affiliations:** 1 Department of Pathology, University of Göttingen, Robert-Koch-Str. 40, 37075 Göttingen, Germany; 2 Department of Oncology, University of Göttingen, Robert-Koch-Str. 40, 37075 Göttingen, Germany

**Keywords:** Malakoplakia, Pleura, Rhodococcus equi, Michaelis-Gutmann-Bodies

## Abstract

Malakoplakia is a disease especially of the urinary tract with typical plaques most frequently observed in the urinary bladder's mucosa. In the context of immunosuppression malakoplakia can also occur in other organs. Some of these extravesical malakoplakias are associated with an infection by Rhodococcus equi, a rare human pathogen well known from veterinary medicine. Here we present the first case of a pleural malakoplakia without lung involvement caused by a proved Rhodococcus equi infection.

## Background

Malakoplakia as a disease of the urinary tract is characterized by typical plaques consisting of accumulated macrophages [1] exhibiting an impaired lysosomal degradation of bacteria, especially E. coli [2,3]. Particularly in immunosuppressed patients malakoplakia can also appear in other organs such as prostate, lung or skin [4]. In some cases of extravesical malakoplakia the patients showed an infection with Rhodococcus equi, a common germ from veterinary medicine, which causes bronchopneumonia in foals [5-7]. Human infections with Rhodococcus equi, however, are very rare and mostly associated with immunosuppression [8]. To our knowledge, affection of the pleura with malakoplakia without lung involvement and simultaneous Rhodococcus equi infection has not been documented previously.

## Case presentation

### Clinical findings

A 60-year-old man was admitted to the hospital because of generalized lymphadenopathy, hepatosplenomegaly and suspicion of pneumonia. Based on lymph node biopsy the diagnosis of T-cell-prolymphocytic-leukemia was confirmed. Allogenic stem cell transplantation was performed with a matched related donor with a HLA-A-mismatch followed by immunosuppression with tacrolimus.

**Figure 1:**
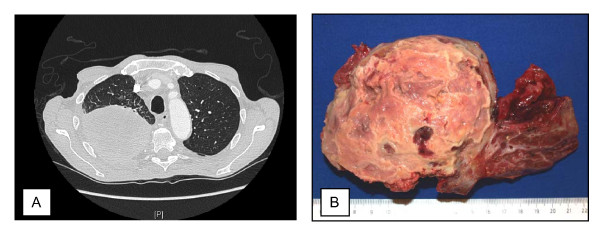
**Radiologic and macroscopic findings**. Axial computer tomography of the chest (A) and the surgical specimen (B) revealed a 12 cm in diameter well circumscribed tumor adherent to pleura and lung.

Three months later the patient was admitted to the hospital with retrosternal pain and a reduced general condition. The clinical examinations revealed pneumonic rales over the right lung, slightly elevated temperature, and elevated infection parameters. Primarily the diagnosis of pneumonia was made and the patient was treated with antibiotics and antimycotics. The following computertomography of the chest revealed a 12 cm in diameter and well circumscribed round mass affecting the right upper lobe of the lung (Figure 1). Differential diagnosis included an organized pneumonia or a malignant tumor. The lesion seemed to repress but not to infiltrate the lung and showed no signs of recovery under therapy.

**Figure 2:**
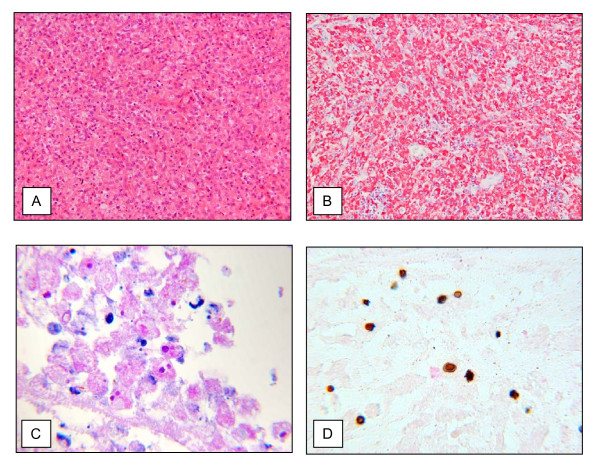
**Microscopic findings**. Histologically and immunohistologically the tumor was composed of CD68 positive macrophages (A: H&E, ×100/B: CD68 immunostaining, ×100) showing cytoplasmatic Michaelis-Gutman bodies (C: PAS, ×400/D: von Kossa, ×400).

Surgery operation confirmed a tumor, which was adherent to the parietal pleura as well as to the lung and which was removed by lobectomy of the right upper lobe. Microbiological examination of tumor tissue revealed a positivity for Rhodococus equi.

### Pathological findings

On macroscopical examination the right upper lobe of the lung showed a 12 cm large tumor connected with the visceral pleura and the lung without infiltration of the lung. The tumor exhibited a gray-white cut surface with central necrotic, disintegrated areas (Figure 1).

Microscopical examination revealed a tumor consisting of a monomorphic cell population, which was predominantly composed of macrophages. Granulomas, multinucleated giant cells or significant cellular atypia could not be observed. The immunohistochemical studies confirmed Ki-M1P/CD68 positive macrophages as the major tumor component. An increased proliferation was not detectable using the Ki67 staining. The macrophages showed intracellular deposits of concentric layers with a bright rim. The latter structures displayed PAS positivity and could be confirmed as calcifications by Kossa staining thus representing Michaelis-Gutmann-Bodies pathognomonic for malakoplakia (Figure 2).

## Conclusion

We present the first case of a pleural malakoplakia caused by a proved Rhodococcus equi infection. The patient suffered from a rare T-cell-prolymphocytic-leukemia and was treated by stem cell transplantation. To prevent rejection, the patient received an immunosuppressive therapy with tacrolimus. Retrospectively, the patient confirmed a contact with horses most probably causing the here described pleural malakoplakia.

Malakoplakia is a rare disease occuring particularly in the area of the urinary tract. It is based on an E. coli infection, usually followed by a macrophage-rich inflammation [1]. During the course of inflammation bacteria can remain and survive within macrophages and form the called Michaelis-Gutmann-Bodies [9]. The macrophages settle in the mucosa of the urinary bladder and form typical plaques. In rare cases usually under suppressed immune status specific pathogens play a major role for the development of malakoplakia [7]. One of these specific pathogens is Rhodococcus equi, a gram-positive rod that is especially known from veterinary medicine [5-7].

The first human infection by Rhodococcis equi was described in 1967 in a drug immunosuppressed patient [10]. Since 1980 the reported cases in the literature of infection with Rhodococcus equi in humans increased up to more than 200 cases till today [11]. This could be due to the increasing occurrence of HIV infection and immunosuppressing therapies in oncology and transplantation medicine. In 30 of these cases a Rhodococcus equi infection with development of malakoplakia could be found, mainly affecting lung tissue without pleural involvement [11,12].

## Consent

Written informed consent was obtained from the patient for publication of this case report and any accompanying images. A copy of the written consent is available for review by the Editor-in-Chief of this journal.

## Competing interests

The authors declare that they have no competing interests.

## Authors' contributions

CLB constructed the manuscript and carried out pathological examination. SN was responsible for the clinical data. SS participated in pathological investigations. HJR was responsible for critical revision of the manuscript and has been involved in drafting it. All authors read and approved the final manuscript.
